# Comparison of Frequentist and Bayesian Generalized Additive Models for Assessing the Association between Daily Exposure to Fine Particles and Respiratory Mortality: A Simulation Study

**DOI:** 10.3390/ijerph16050746

**Published:** 2019-03-01

**Authors:** Xin Fang, Bo Fang, Chunfang Wang, Tian Xia, Matteo Bottai, Fang Fang, Yang Cao

**Affiliations:** 1 Unit of Biostatistics, Institute of Environmental Medicine, Karolinska Institutet, Stockholm 17177, Sweden; fx63783985@hotmail.com (X.F.); matteo.bottai@ki.se (M.B.); 2 Division of Vital Statistics, Shanghai Municipal Center for Disease Control and Prevention, Shanghai 200336, China; fangbo@scdc.sh.cn (B.F.); wangchunfang@scdc.sh.cn (C.W.); 3 Institute of Health Information, Shanghai Municipal Center for Disease Control and Prevention, Shanghai 200336, China; xiatian@scdc.sh.cn; 4 Department of Medical Epidemiology and Biostatistics, Karolinska Institutet, Stockholm 17177, Sweden; fang.fang@ki.se; 5 Clinical Epidemiology and Biostatistics, School of Medical Sciences, Örebro University, Örebro 70182, Sweden

**Keywords:** Bayesian statistics, generalized additive model, time-series analysis, fine particulate matter, respiratory mortality

## Abstract

*Objective:* To compare the performance of frequentist and Bayesian generalized additive models (GAMs) in terms of accuracy and precision for assessing the association between daily exposure to fine particles and respiratory mortality using simulated data based on a real time-series study. *Methods*: In our study, we examined the estimates from a fully Bayesian GAM using simulated data based on a genuine time-series study on fine particles with a diameter of 2.5 μm or less (PM_2.5_) and respiratory deaths conducted in Shanghai, China. The simulation was performed by multiplying the observed daily death with a random error. The underlying priors for Bayesian analysis are estimated using the real world time-series data. We also examined the sensitivity of Bayesian GAM to the choice of priors and to true parameter. *Results*: The frequentist GAM and Bayesian GAM show similar means and variances of the estimates of the parameters of interest. However, the estimates from Bayesian GAM show relatively more fluctuation, which to some extent reflects the uncertainty inherent in Bayesian estimation. *Conclusions*: Although computationally intensive, Bayesian GAM would be a better solution to avoid potentially over-confident inferences. With the increasing computing power of computers and statistical packages available, fully Bayesian methods for decision making may become more widely applied in the future.

## 1. Introduction

With accelerated urban development and modernization, air pollution is worsening and its impact on human health has been the main research topic in developing countries [1]. Air pollutants include gaseous pollutants and particulate matter (PM). Studies have shown that PMs with an aerodynamic diameter of 2.5 µm or less (referred to as PM_2.5_ or fine particles) have a greater impact on human health [2,3]. The impact of both long term (chronic) and short term (acute) exposure to PMs has been widely studied, with the former being most often estimated from cohort studies [4] and the latter from ecological time-series studies [5]. However, cohort studies are expensive and time-consuming to implement due to the long follow-up period required for collecting individual-level pollution and disease data [6]. This has led to the use of spatiotemporal ecological study design in this field [7,8], which takes advantage of routinely collected data [5] such as data from the air quality monitoring (AQM) stations in Beijing and the Causes of Death Registry (CDR) of the Chinese Centers for Disease Control and Prevention [9]. Although causal inference is an important problem in time-series studies due to the difficulty of selecting an appropriate regression model that can be well fitted to the data, they contribute to and independently corroborate the body of evidence provided by cohort studies [10].

The semiparametric Poisson regression has been widely used for time-series analyses of air pollution and health, which uses daily mortality or morbidity counts as the outcome, linear terms to measure the percentage of increase in the outcome associated with elevations in air pollution levels, and smooth functions of time and weather variables to adjust for time-varying confounders [11]. Generalized linear models (GLMs) with parametric splines (e.g., natural cubic splines) [12] or generalized additive models (GAMs) with nonparametric splines (e.g., smoothing splines or locally weighted smoothers or (LOESS) [13] are used to estimate effects associated with exposure to air pollution while accounting for smooth fluctuations in outcome that confound the estimated effects of pollution. GAM and GLM can be applied in similar situations, but they serve different analytic purposes. GLM emphasizes estimation and inference for the parameters of the model, while GAM focuses on exploring data non-parametrically. GAM is more suitable for exploring the data and visualizing the relationship between the dependent variable and the independent variables [14].

The basic form of GAM applied in air pollution and health studies may be expressed using Equation (1) [11]:

(1) $$log\left(E\left(Y_{t}\right)\right)=\beta_{0}+\beta_{l}X_{t-l}+S\left(t\right)+S\left(weather\right)+\phi \cdot DOW_{t}$$

where *Y_t_* is count of daily mortality or morbidity, *β*_0_ denotes the intercept, *t* indicates calendar day, *X_t_* are daily concentrations of the studied air pollutant, i.e., PM_2.5_ in our study, *l* is the lag time of the pollution exposure (which is generally restricted to one to seven days for acute effects), *S*(*t*) denotes a smooth function of a covariate (calendar day or meteorological variables such as temperature and humidity). The smooth functions are usually constructed using LOESS, smoothing splines or natural cubic splines. *ϕ* is the vector of the regression coefficients associated with vector *DOW_t_* (indicating the 7 days of a week) for the *t*th day. *β_l_* is the parameter of interest describing the change in the logarithm of the average mortality count over population per unit of change in *X_t_*_−*l*_, which is generally interpreted as the percentage of increase in mortality for every 10 units or a standard deviation (SD) or interquartile range (IQR) of increase in ambient concentrations of the studied air pollutant at lag *l*. The reason that the model is called semiparametric is that it assumes a linear relation with *X_t_*_−*l*_ and unknown functional relations with time and weather variables.

The conventional algorithm for fitting GAM (hereinafter called frequentist GAM) is the backfitting algorithm [15] and the corresponding robust estimation method has also been developed [16]. A disadvantage of backfitting is that it is difficult to integrate with the estimation of the degree of smoothness of the model terms, so that in practice the user must set these, or select between a modest set of pre-defined smoothing levels. Although the degree of smoothness can be estimated as part of model fitting using generalized cross-validation or by restricted maximum likelihood when the smooth components are represented using smoothing spline, it carries a computational cost [17]. The computationally efficient approaches such as fully Bayesian method have thus been developed in recent years [18].

Although there are some applications [19,20,21,22,23] of Bayesian GAM analyses in recent years, few of them compared the performance of frequentist and Bayesian GAMs in terms of accuracy and precision. In our study, we examined the estimates from a fully Bayesian GAM using simulated data based on underlying ‘true’ parameters from a genuine time-series study on PM_2.5_ and respiratory deaths conducted in Shanghai, China.

## 2. Methods

### 2.1. Real World Data

The data of our study were from the Swedish-Chinese joint project Assessing Variability in the Short-term Impact of Air Pollution and Extreme Weather Conditions on Non-accidental Deaths in Shanghai during 2012–2014. The study was approved by the Ethical Review Committee of the Shanghai Municipal Center for Disease Control and Prevention (SCDC), China (approval number: SCDC2016-08).

Briefly, daily average PM_2.5_ concentrations between 1 January 2012 and 31 December 2014 were obtained from one fixed air quality monitor of the Shanghai Meteorological Bureau and one monitor from the U.S. Consulate General in Shanghai, China. Daily mortality data during the corresponding time period for all the permanent residents in Shanghai were obtained from the Causes of Death Registry of the SCDC. The causes of death were coded according to the International Classification of Diseases Codes, version 10 (ICD-10). Deaths for respiratory diseases (ICD-10 codes J00-J99) were retrieved. Citywide daily meteorological data including temperature, relative humidity, barometric pressure, wind speed, precipitation, and sunshine time were retrieved from the Shanghai Meteorological Bureau.

**Figure 1:**
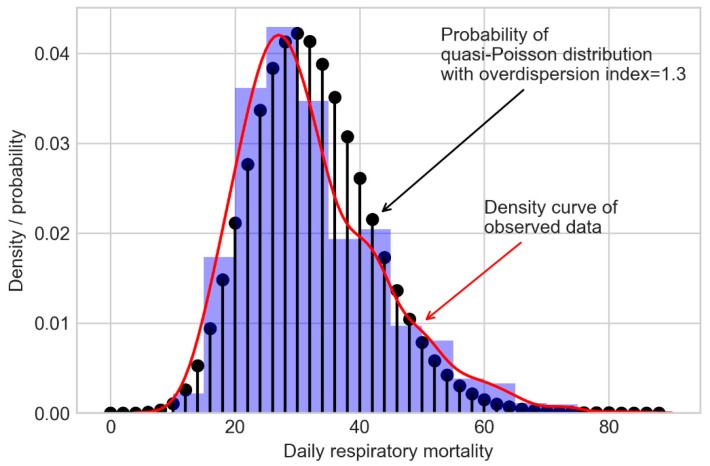
Distribution of observed daily respiratory mortality (the red curve) and theoretical distribution quasi-Poisson distribution (the black vertical lines, mean = 32, overdispersion index = 1.3).

The distribution of the observed daily respiratory mortality and the theoretical quasi Poisson distribution with the same mean and an overdispersion index = 1.3 are shown in Figure 1. The observed data showed a few deviations from the theoretical distribution. Therefore, a Poisson regression model is suitable for our data.

**Figure 2:**
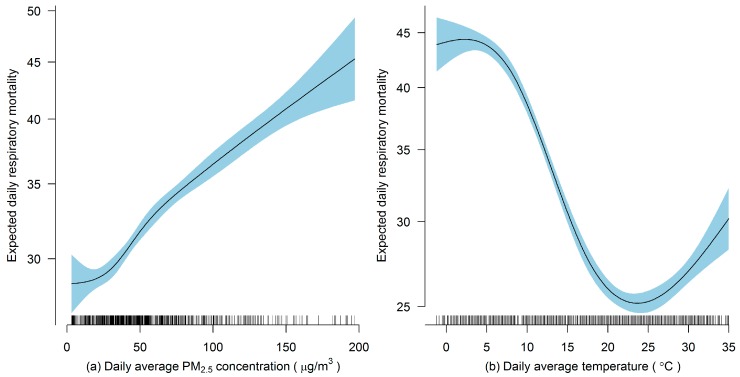
Smoothing plots of (**a**) daily respiratory mortality against daily average PM_2.5_ concentration and (**b**) daily average temperature.

To illustrate the relationship between respiratory mortality and PM_2.5_ pollution and meteorological variables, we used the daily respiratory mortality and the corresponding average PM_2.5_ concentration and the average temperature in lag 1 as an example (Figure 2). In general, daily respiratory mortality was positively related to PM_2.5_ concentration and an approximately linear relationship was found. A similar relationship was found for PM_2.5_ concentrations at other lags. However, an apparent reversed sigmoid relationship was found between daily respiratory mortality and daily average temperature. A nonlinear relationship was also found for other meteorological variables. Therefore, a GAM with linear components for PM_2.5_ concentrations and nonlinear components for weather conditions and time trend is suitable for our study.

### 2.2. Fully Bayesian Generalized Additive Model

A fully Bayesian approach for modeling and inference within GAM requires prior assumption for unknown function *S*(*t*). Several alternatives have been recently proposed to specify smoothness prior for continuous covariates or time trends, such as random walk priors or more generally autoregressive priors [18,24], Bayesian P(enalized)-splines [25], and Bayesian smoothing splines [26]. Bayesian P-splines were used in our study because it has the advantage of allowing for simultaneous estimation of smooth functions and smoothing parameters. Moreover, it can easily be extended to more complex formulations [25]. The method assumes that an unknown smooth function *S_j_*(*x_j_*) of a covariate *x_j_* can be approximated by a polynomial spline of degree *ν* defined on a set of equally spaced knots $x_{j}^{min}=\zeta_{0}<\zeta_{1}<\cdots <\zeta_{d-1}<\zeta_{d}=x_{j}^{max}$ within the domain of *x_j_*. Such a spline can be written in terms of a linear combination of *M_j_*(= *d* + *ν*) B-spline basis functions *B_m_*, i.e.,

(2) $$S_{j}\left(x_{j}\right)=\overset{M_{j}}{\underset{m=1}{\sum }}\beta_{j,m}B_{m}\left(x_{j}\right)$$

and $\beta_{j}={\left(\beta_{j,1},\cdots ,\beta_{j,M_{j}}\right)}^{\prime }$ corresponds to the vector of unknown regression coefficients. The functions *B_m_* are only positive with an area spanned by *ν* + 2 knots, which is essential for the construction of the smoothness penalty for P-splines. The estimation of *S_j_*(*x_j_*) is thus reduced to the estimation of the vector of unknown regression coefficients *β_j_* from the data. To choose the number of knots, a moderately large number of equally spaced knots (around 10 knots per time interval) as suggested by Eilers and Marx [27] is flexible enough to capture the variability of the data. In the Bayesian approach, penalized splines are introduced by replacing the different penalties with their stochastic analogues, i.e., the first or second order random walk priors for the regression coefficients. First order random walk prior for equidistant knots is given by [28]:

(3) $$\beta_{j,m}=\beta_{j,m-1}+u_{j,m},\;m=2,\;\cdots ,\;M_{j}$$

and a second order random walk by:

(4) $$\beta_{j,m}=2\beta_{j,m-1}-\beta_{j,m-2}+u_{j,m},\;m=3,\;\cdots ,\;M_{j}$$

with Gaussian errors $u_{j,m}\sim N\left(0,\;\tau_{j}^{2}\right)$ and diffuse priors $p\left(\beta_{j,1}\right)\propto const$, or $p\left(\beta_{j,1}\right)\;\mathrm{and}\;p\left(\beta_{j,2}\right)\;\propto const$, for initial values, respectively.

When we define the unknown function evaluation *S_j_* as the matrix product of a design matrix *ψ_j_* and a vector of unknown parameters *β_j_*, i.e.,

(5) $$S_{j}=\psi_{j}\beta_{j},\;j=1,\;\cdots ,p$$

then we obtain the predictor in Equation (1) as

(6) $$log\left(E\left(Y_{t}\right)\right)=\beta_{0}+\beta_{l}X_{t-l}+\sum_{j=1}^{p}\psi_{j}\beta_{j}+\phi \cdot DOW.$$

Depending on the above parameterization of the model, the posterior for fully Bayesian inference is given by:

(7) $$p(\beta_{0},\;\beta_{l},\;\beta_{1},\;\cdots ,\;\beta_{p},\;\tau_{1}^{2},\cdots ,\tau_{p}^{2},\;\phi |y)\propto L\left(y,\beta_{0},\beta_{l},\beta_{1},\;\cdots ,\;\beta_{p},\;\phi \right)\overset{p}{\underset{j=1}{\prod }}\left(p(\beta_{j}|\tau_{j}^{2})p\left(\tau_{j}^{2}\right)\right)$$

where *L* denotes the likelihood which is the product of individual likelihood contributions.

In the fully Bayesian approach, parameter estimates are obtained by drawing random samples from the posterior Equation (7) via Markov Chain Monte Carlo (MCMC) simulation techniques. More details about the fully Bayesian inference can be found in Fahrmeir and Lang [18] and Brezger and Lang [29].

### 2.3. Estimation of True Parameters

Although we focused on the effect of PM_2.5_ concentrations in day lag 1 in the study, to exclude the effects from other lag days, the ‘true’ parameters were estimated using the frequentist distributed lag GAM instead of the basic model aforementioned. The distributed lag model associates health outcomes in a given day with PM_2.5_ concentrations in several earlier days by replacing *β_l_X*_*t*−*l*_ in (1) with:

(8) $$\sum_{l=1}^{L}\beta_{l}X_{t-l}\;\mathrm{or}\;\theta \sum_{l=1}^{L}\eta_{l}X_{t-l},\;\sum_{l=1}^{L}\eta_{l}=1$$

where *θ* measures the cumulative effect of PM_2.5_ during the days, and *η_l_* measures the contribution of the lagged exposure *X_t_*_−*l*_ to *θ* [30,31]. To reduce the computational work, we introduced only one smoothness term i.e., seasonal trend in the model. The nonlinear effects from meteorological variables were examined using a categorical variable for six synoptic weather types (SWTs) [32]. The final model may be expressed as:

(9) $$log\left(E\left(Y_{t}\right)\right)=\beta_{0}{+\beta }_{1}\cdot {\mathrm{lag}1}_{t}{+\beta }_{2}\cdot {\mathrm{lag}2}_{t}+\cdots {+\beta }_{7}\cdot {\mathrm{lag}7}_{t}+S(t)+\phi_{W}\cdot W_{t}+\phi \cdot {DOW}_{t}$$

where *β_l_* (*l* = 1, …, 7) approximate to the percentage increase in deaths associated with the corresponding explanatory variables, i.e., lag1*_t_*–lag7*_t_*, the daily PM_2.5_ concentrations of lag 1–7 days. Frequentist *S*(*t*) was realized using cubic B-splines with in total 18 knots (6 knots per year). $W_{t}$ denotes the vector of the six SWTs, i.e., hot dry, warm humid, cold dry, moderately dry, moderately humid, and cold humid weather types. The SWTs are defined using 15 routinely monitored meteorological variables through principal component analysis and K-means clustering method. The details of the methods were published elsewhere [33].

In total, 35,135 respiratory deaths occurred during the study period between 1 January 2012 and 31 December 2014 in Shanghai. Mean and median daily deaths were 32 and 30, respectively. The descriptive statistics of daily respiratory deaths, ambient PM_2.5_ concentrations, and major meteorological variables are shown in Table 1.

The parametric coefficients of the covariates derived from the frequentist GAM are shown in Table 2.

In our study, we focused on the acute effect of PM_2.5_ concentrations on respiratory mortality, i.e., *β*_1_ of lag 1 day in Equation (9). It is 0.0049 and statistically significant (corresponding to 5.37 excessive deaths per day or 1960 excessive deaths per year). The standard error (SE) of *β*_1_ is 0.0017. The unit of PM_2.5_ concentrations is 10 μg/m^3^ in the analysis, therefore *β*_1_ can be explained as that per 10 μg/m^3^ increase in PM_2.5_ concentration of lag 1 day is associated with approximately 0.49% increase in daily respiratory deaths. The result is consistent with the findings from two meta-analyses [34,35] and a large observational study performed in China [36], where the corresponding percent increase in respiratory mortality ranged from 0.29% to 0.75%.

### 2.4. Simulation

**Figure 3:**
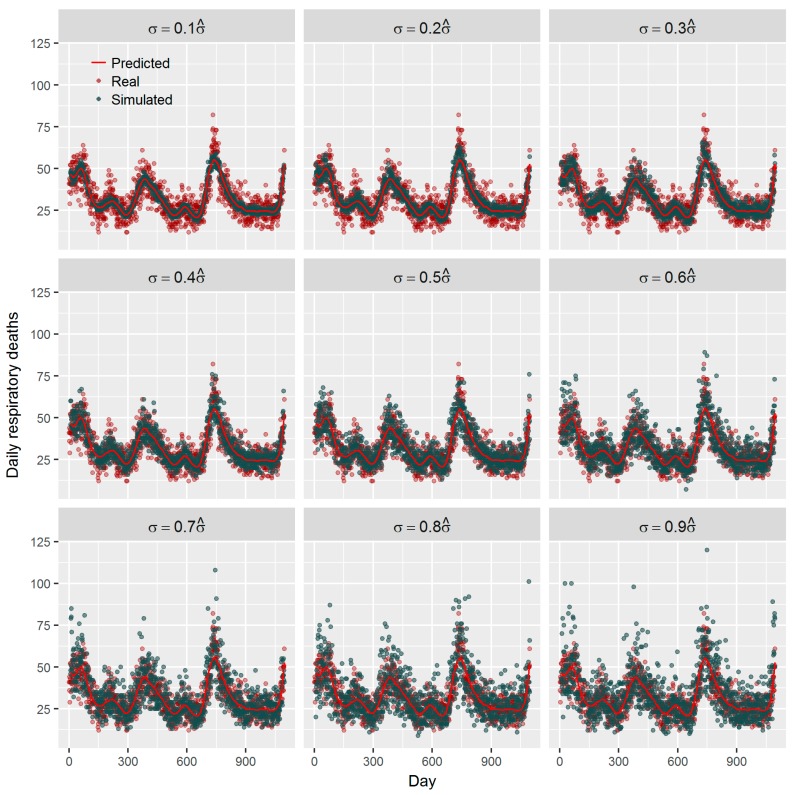
Examples of simulated time-series of daily respiratory deaths with different random noises.

To assess the estimation of the fully Bayesian GAM approach compared with the realistic situations, we conducted a simulation study. In our simulation, the estimates in Table 2 derived from the real world data were used as ‘true’ parameters. We used the predicted daily deaths $\hat{Y_{t}}$ based on Equation (9) as the mean daily deaths, then simulated daily death $Y_{t}^{\prime }$ by multiplying $\hat{Y_{t}}$ by a random error *e^ε^*:

(10) $$Y_{t}^{\prime }=\hat{Y_{t}}\cdot e^{\varepsilon },\;\varepsilon \sim N\left(0,\;\sigma^{2}\right)$$

where *ε* follows a distribution from exponential family and we applied normal distribution here. The simulation framework ensures that the same concurvity will exist between the simulated mortalities and the observed ones. By changing *σ* we may introduce different ‘noise’ in mortality to simulate the effects of unobserved confounders. In our simulation, the changing of the *σ* was achieved by multiplying $\hat{\sigma }$, the SD of logarithmic daily deaths, by a factor *γ*, i.e., $\sigma =\gamma \hat{\sigma }$. By selecting different random seeds, we may generate different time-series using random number generator in any statistical software. Figure 3 shows nine simulated time-series of daily respiratory deaths for *γ* = 0.1, 0.2, …, 0.9. We can see when *γ* is equal to 0.4 or 0.5 the simulated data are most approximate to the real world data.

**Figure 4:**
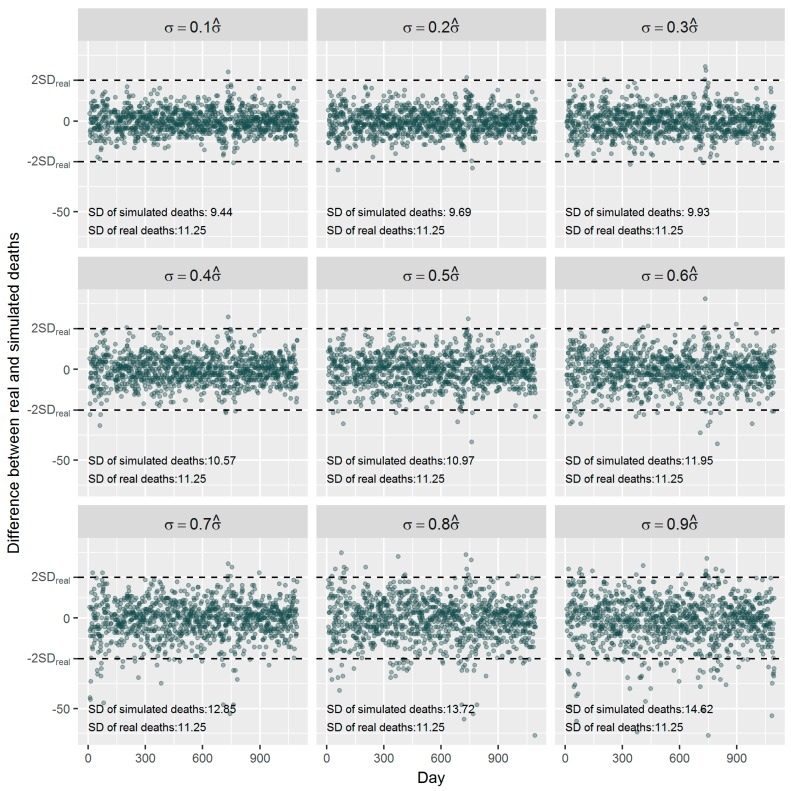
Examples of the difference between the real and the simulated daily respiratory deaths with different random noises.

Figure 4 shows the difference between the simulated data and the real world data. Almost all the differences were within the range of ±2SD of the real data when *γ* is between 0.1 and 0.9. The larger *γ* the larger difference presents, which means more ‘noise’ being included in the simulated data. When *γ* is equal to 0.5 the difference between SDs of the simulated data and the real world data (10.97 vs. 11.25) is the smallest. Therefore, we used the simulated data with *γ* ≥ 0.5 in the following analyses to include relatively larger uncertainty for our estimation.

## 3. Results

### 3.1. Comparison of the Results from Frequentist GAM and Bayesian GAM

**Figure 5:**
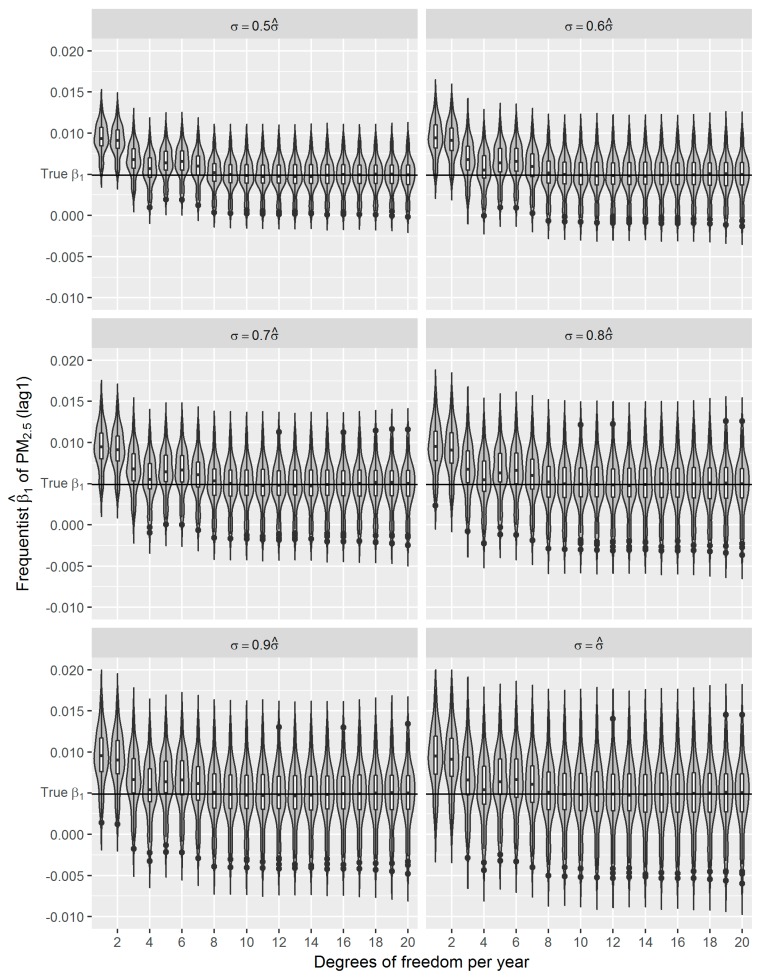
Distributions of frequentist ${\hat{\beta }}_{1}$s for simulated data with different random noises (*σ =* 0.5 − 1.0$\hat{\sigma }$) and degrees of freedom (1–20 per year) for *S*(*t*); the true *β*_1_ = 0.0049.

First, we set *γ* = 0.5, 0.6, …, 1.0 to generate six sets of simulated respiratory mortality data, where each set included 2000 time-series. Then we run the frequentist GAMs using simulated daily mortality as the dependent variable. We set the degrees of freedom (*df*) for *S*(*t*) from 1 to 20 per year in our models. For each *df*, we ran the frequentist GAM using 100 simulated time-series. In total, 12,000 ${\hat{\beta }}_{1}$s (from 6 *γ*s × 20 *df*s × 100 time-series) were derived. Distributions of the estimated ${\hat{\beta }}_{1}$s against the true *β*_1_ are shown using a violin plot combined with a box plot in Figure 5.

In general, although the variance of ${\hat{\beta }}_{1}$s tends to increase as the *df* for *S*(*t*) is increased, the decrease in bias is far more dramatic, i.e., the greater *df* the closer mean of ${\hat{\beta }}_{1}$s approach to the true *β*_1_. The bias in the estimates is only serious for *df* ≤ 4 per year. With sufficient *df* to represent the smoothness of the nonparametric nonlinear trend leads to an asymptotically unbiased estimate of ${\hat{\beta }}_{1}$ [37]. The apparent decrease in the bias of ${\hat{\beta }}_{1}$s with increasing *df* is explained by Daniels et al. [38], Rice [39] and Speckman [40]. As we expected, the increased noise in the simulated data results in a larger variance of ${\hat{\beta }}_{1}$s (Figure 5 and Table 3), which is approximate to 2*γ* × SE(*β*_1_) or 2*γ* × 0.0018.

**Figure 6:**
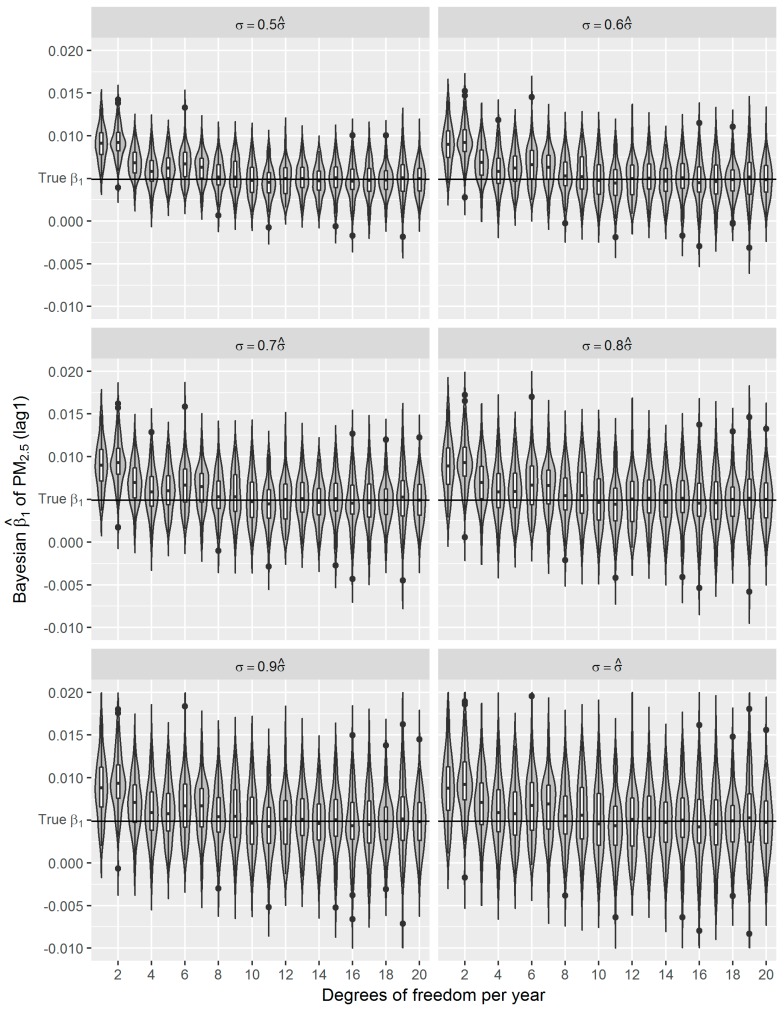
Distributions of Bayesian ${\hat{\beta }}_{1}$s for simulated data with different noises (*σ =* 0.5 − 1.0$\hat{\sigma }$) and degrees of freedom (1–20 per year) for *S*(*t*); the true *β*_1_ = 0.0049.

The Bayesian GAM analyses were conducted in software SAS 9.4 and R version 3.41 with MCMCpack package [41]. We used 1000 burn-in iterations and 2000 iterations after burn-in for Markov chains. Although 10,000 iterations after burn-in are usually suggested, we did not find noticeable differences in the estimates between 10,000 iterations and 2000 iterations. The latter reduced however the computation time significantly. For comparison purpose, we also used *df*s ranged from 1 to 20 per year for the Bayesian P-splines to control for smoothness. By default we used non-informative uniform priors for the coefficients and the derived estimates are quite similar to those derived from frequentist GAMs. Although the Bayesian ${\hat{\beta }}_{1}$s appear more fluctuated around the true *β*_1_ (Figure 6), their SDs are comparable to those of their frequentist counterparts (Table 4).

### 3.2. Sensitivity of Bayesian GAM to Choice of Prior Mean and Variance

**Figure 7:**
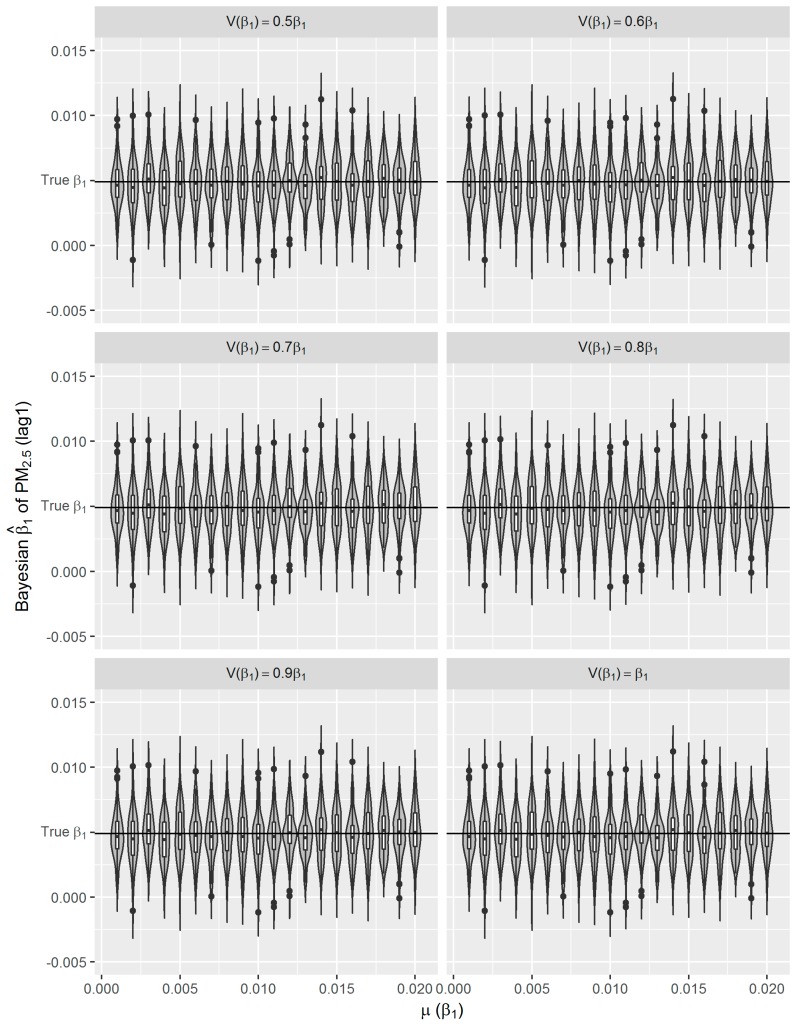
Distributions of Bayesian ${\hat{\beta }}_{1}$s from simulated data with *σ =* 0.5$\hat{\sigma }$, the true *β*_1_ = 0.0049; in Bayesian GAM analyses *df* = 8 for *S*(*t*), V(*β*_1_) equal to *γβ*_1_, where *γ* = 0.5, 0.6, …, 1.0, and µ(*β*_1_) ranging from 0.001 to 0.020 by 0.001.

One of the most important (however also controversial) features of Bayesian method is that they can integrate prior knowledge and observed data in their inference. The posterior is a compromise between prior and likelihood. In the second simulation, we investigated the impact of informative priors rather than non-informative uniform prior on the posterior ${\hat{\beta }}_{1}$. We simulated the time-series of daily respiratory mortality with a fixed σ = 0.5$\hat{\sigma }$ and true *β*_1_ = 0.0049. In total, 12,000 time-series were generated. For Bayesian GAM analyses, we used a normal prior for *β*_1_, and set the varied prior mean µ(*β*_1_) ranging from 0.001 to 0.020 by 0.001, and varied prior variance V(*β*_1_) equal to *γβ*_1_, where *γ* = 0.5, 0.6, …, 1.0. For each combination of µ(*β*_1_) and V(*β*_1_), we did 100 Bayesian analyses. To get unbiased estimates with fewer computation task, we set the *df* for splines to eight per year. The distribution of Bayesian estimates (${\hat{\beta }}_{1}$s) are shown in Figure 7. The mean of ${\hat{\beta }}_{1}$s is fluctuated but closely around the true *β*_1_ for different µ(*β*_1_). These is no noticeable difference among the means of ${\hat{\beta }}_{1}$s derived from different V(*β*_1_) (Figure 7 and Table 5). The SD of ${\hat{\beta }}_{1}$s is not sensitive to the V(*β*_1_) (Table 5).

### 3.3. Sensitivity of Bayesian GAM to True Parameter

In our third simulation study, we artificially set the *σ* = 0.5$\hat{\sigma }$ and ‘true’ *β*_1_ ranged from 0.001 to 0.020 to generate 20 sets of simulated daily respiratory deaths, while kept the other coefficients in Equation (9) unchanged.

**Figure 8:**
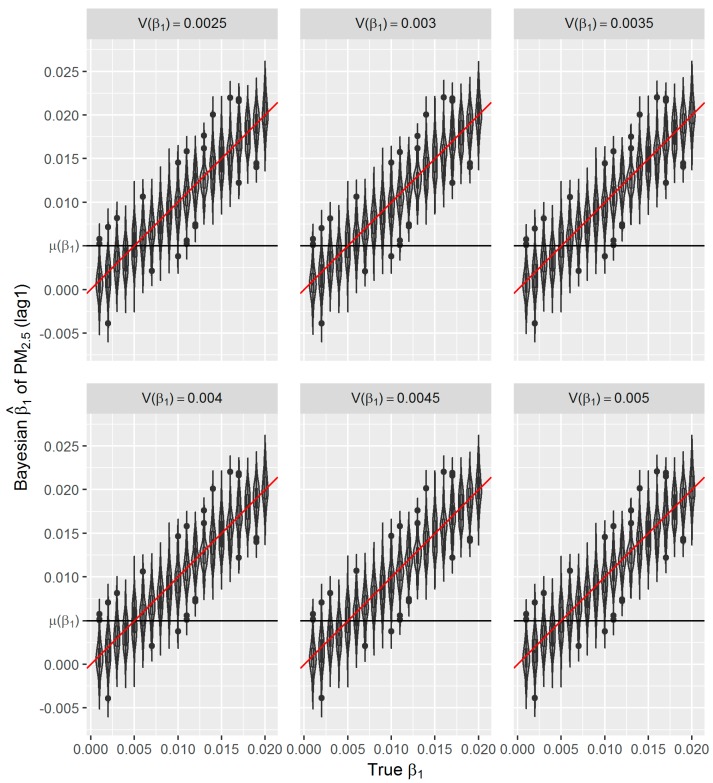
Distributions of Bayesian ${\hat{\beta }}_{1}$s from simulated data with *σ =* 0.5$\hat{\sigma }$, true *β*_1_ = 0.001 to 0.02 by 0.001; in Bayesian GAM analyses *df* = 8 for *S*(*t*), µ(*β*_1_) = 0.005 and V(*β*_1_) = 0.0025, 0.003, 0.0035, 0.004, 0.0045 and 0.005.

In Bayesian estimation, we used a normal prior for *β*_1_ with a fixed mean µ(*β*_1_) = 0.005 but varied V(*β*_1_) = 0.5, 0.6, …, 1.0 times of µ(*β*_1_), i.e., 0.0025, 0.003, 0.0035, 0.004, 0.0045 and 0.005. For each combination of *β*_1_ and V(*β*_1_), we generated 100 simulated time-series. Therefore, for each prior *β*_1_, we did 600 Bayesian GAM analyses (100 × 6 variances). The estimates by the 20 true *β*_1_s and 6 varied variances were shown in Figure 8 and Table 6.

We can see that the mean of the estimated ${\hat{\beta }}_{1}$s is only sensitive to the underlying true *β*_1_ and is almost not affected by the prior µ(*β*_1_). Due to the small coefficients, the difference between means and SDs of the estimated ${\hat{\beta }}_{1}$s can only be seen in the fifth or sixth decimal digit. The bivariate linear regression analysis between the true *β*_1_s and the Bayesian ${\hat{\beta }}_{1}$s in Table 6 reveals that all the coefficients are almost equal to 1 and all the R are larger than 0.99, which indicates the high precision and accuracy of the Bayesian estimates.

## 4. Discussion

In the presented study, we evaluated the performance of frequentist and fully Bayesian GAM approaches in a time-series study on the relationship between daily exposure to PM_2.5_ and respiratory mortality. According to our estimates, per 10 μg/m^3^ increase in PM_2.5_ concentration of lag 1 day is associated with an approximately 0.49% increase in daily respiratory deaths in Shanghai between 2012 and 2014, which is consistent with the results from other studies conducted in China [9,36]. Using the estimated effect as true parameter, we compared the frequentist GAM and Bayesian GAM based on simulation. Both frequentist GAM and Bayesian GAM show the similar mean estimates of the interested parameters. However, the estimates from frequentist GAM showed relatively less fluctuation (Figure 5 and Figure 6), which to some extent reflects the over-confident inferences embedded in this method. Regarding the accuracy and precision of the estimates, both methods gave mean estimates close to the true parameter with comparable confidence intervals. It means that Bayesian GAM might be an ideal alternative to the conventional frequentist GAM. Our simulation study also indicated that when the underlying parameter was true, the informative normal priors had no noticeable influence on the Bayesian estimate (Figure 7), which was only sensitive to the underlying true parameter (Figure 8). The reason might be the large number of data that we have and the posterior is dominated by the data rather than the prior. 

As a flexible extension of GLM introduced by Hastie and Tibshirani [42], GAM can estimate both linear trends from parametric components and nonlinear trend from any general nonparametric components during the fitting. It has been widely used in time-series studies on air pollution and health effects, controlling for daily variations in meteorological conditions and seasonal trends. The original GAM fitting method estimated the smooth components of the model using non-parametric smoothers, such as smoothing splines or LOESS, via the backfitting algorithm [42]. By iteratively smoothing partial residuals, backfitting provides a general module to estimate the *S_j_* terms that are capable of using a wide variety of smoothing methods. The computational cost issue of full spline method has been addressed recently by using Markov random fields to find sparse representations of the smooths, which can be viewed as an empirical Bayesian method [43]. An alternative approach with particular advantages in high dimensional settings is to use boosting, which typically requires bootstrapping for uncertainty quantification [44,45].

Although frequentist GAM gives a rich family of models that have been widely applied, a crucial problem with GAM is the choice of the number and the position of the knots, in terms of analytical tractability. A small number of knots may result in insufficient flexibility to capture the variability of the data while a large one may lead to overfitting. P-splines approach makes a more parsimonious parameterization possible, which is of particular advantage in a Bayesian framework where inference is based on MCMC techniques [25]. By taking into account the complete likelihood surface rather than plugging in the maximum likelihood estimate of the covariance structure, the approach provides the posterior distributions of the quantities of interest, such as ‘the true parameter has a probability of 0.95 of falling in a 95% credible interval (CI)’, which is more interpretable. Unlike frequentist methods, which provided one estimate for each model parameter, Bayesian methods may provide, for each parameter, a sample of thousands of MCMC estimates from the simulated posterior distribution of the parameter. The reported posterior mean and posterior distribution are the corresponding summaries of the marginal posterior distribution of the parameter.

Due to recent developments in MCMC algorithms, software, and hardware, we can now use MCMC methods to analyze complex models that would have been impossible only a few decades ago [46]. However, the Bayesian GAM is only available in a few R packages such as gammSlice [47], R2BayesX [48], and spikeSlabGAM [49], which limits its application in a variety of scientific fields. In gammSlice Pham et al. used the slicing sampling method [50] for GAM fitting and inference within a Bayesian framework [47]. R2BayesX supports similar models to those in gammSlice. Scheipl used spike-and-slab type prior distributions on the spline coefficients [49]. Especially, Klein et al. proposed a general class of Bayesian GAM for count data within the GAM framework for location, scale, and shape where semiparametric predictors can be specified for several parameters of a count data distribution [21]. 

There are some limitations to our study. First, we did not impose any structure on the relationship of the coefficients of the lagged PM_2.5_ concentrations with each other. However, multicollinearity among the lagged independent variables often arises, leading to high variance of the coefficient estimates. We plan to address this problem by constraining the *β_l_* to be a simple function of the lag number using a structured finite distributed lag model in the future [51]. This problem can also be addressed by a time-scale model which estimates the association between daily smooth variations of pollution and health outcomes by replacing *β_l_X_t_*_−*l*_ in Equation (1) with:

(11) $$\sum_{k=1}^{K}\beta_{k}W_{kt}$$

where *W*_1*t*_, …, *W_kt_*, …, *W_Kt_* is a set of predictors obtained by applying a wavelet analysis or Fourier analysis to *X_t_* to satisfy orthogonality, such that $\sum_{k=1}^{K}W_{kt}=X_{t}$ [52,53,54]. The parameter *β_k_* measures the logarithmic relative rate of the health outcome for increasing air pollution at time scale *k*. Time scales of interest include short-term variations within several days and long-term variations within one to two months because it is believed that any effects are dominated by seasonal confounding beyond two months [54]. Other tools such as auto-regressive moving average model or vector auto-regressive model could be also appropriate [55]. Second, our simulation framework did not address the issue of measurement error in the covariates. Because such error can in some situations attenuate the estimated effects, it may be useful in the future to employ a more elaborate simulation framework to incorporate the measurement error. Third, our model did not model interaction between PM_2.5_ and other covariates yet. The concentration-response relationship between PM_2.5_ and respiratory deaths was assumed to be approximately linear, but this might not be true in very low or very high PM_2.5_ concentrations. The two limitations can be addressed using varying coefficient model (VCM), where linear $\beta_{l}X_{t-l}$ is replaced by *S*(*X_t_*_−*l*_)*z_l_*. Estimation of VCM poses no further difficulties since only the *β_l_* in Equation (7) has to be redefined by *S*(*X_t_*_−*l*_) multiplying with *Z_l_*. Furthermore, we acknowledge that our study is based on simulated data, and the difference in the results between the frequentist and Bayesian methods is small and might be biased towards a Bayesian approach because of the predefined parameters. Therefore, the evidence to advocate the Bayesian approach over the frequentist one has yet been strong, and a confirmation study using real-world data is needed to address these issues in the future.

## 5. Conclusions

Our simulation study indicates that fully Bayesian GAM may generate an accurate and precise estimations as conventional frequentist GAM does while revealing potential uncertainty that frequentist GAM could not detect. Although computationally intensive, Bayesian GAM would be a better solution to avoid over-confident inferences potentially seen in a frequentist GAM. With the increasing computing power and available statistical packages, fully Bayesian methods may see wider applications in decision-making processes.

## Figures and Tables

**Table 1:** Descriptive statistics of daily respiratory deaths, ambient PM_2.5_ concentrations, and major meteorological variables.

Variable	*n*	Mean	SD	Min	*P*_25_	*P*_50_	*P*_75_	Max
Respiratory deaths	1096	32	11	12	24	30	38	82
log(Respiratory deaths)	1096	3.41	0.34	2.48	3.18	3.40	3.64	4.41
PM_2.5_ (μg/m^3^)	1091	55.0	38.6	3.0	29.4	45.5	68.7	447.5
Temperature (°C)	1096	17.2	9.0	−1.2	8.8	18.2	24.3	35.0
Barometric Pressure (kPa)	1096	101.6	0.9	99.5	100.8	101.6	102.3	103.8
Relative Humidity (%)	1096	70.3	12.6	30	62	72	80	98
Wind speed (m/s)	1096	2.8	1.0	0.6	2.1	2.7	3.4	8.6
Precipitation (mm)	1096	3.3	10.4	0	0	0	1.1	195.3
Sunshine (h)	1096	4.7	4.0	0	0	4.8	8.2	12.9

SD, standard deviation; *P_x_*, the *x*th percentile.

**Table 2:** Parametric coefficients of the covariates in Equation (9).

Coefficient	Estimate	Standard Error	*z* Value	*Pr*(>|*z*|)
*β*_0_	3.9666423	0.1214331	32.665	2*e*-16
*β*_1_	0.0049014	0.0016902	2.900	0.00373
*β*_2_	−0.0020338	0.0018396	−1.106	0.26892
*β*_3_	0.0024222	0.0018156	1.334	0.18219
*β*_4_	−0.0000745	0.0018219	−0.041	0.96738
*β*_5_	0.0004227	0.0018279	0.231	0.81712
*β*_6_	0.0006673	0.0018274	0.365	0.71500
*β*_7_	−0.0004166	0.0018230	−0.229	0.81922
*ϕ_W_*_1_	Reference			
*ϕ_W_*_2_	−0.0612972	0.0220647	−2.778	0.00547
*ϕ_W_*_3_	−0.0780534	0.0320905	−2.432	0.01500
*ϕ_W_*_4_	−0.0494911	0.0267129	−1.853	0.06392
*ϕ_W_*_5_	−0.0085775	0.0274555	−0.312	0.75473
*ϕ_W_*_6_	−0.0603464	0.0319503	−1.889	0.05892
*ϕ*_0_	Reference			
*ϕ*_1_	−0.0094768	0.0202340	−0.468	0.63953
*ϕ*_2_	0.0010382	0.0201531	0.052	0.95891
*ϕ*_3_	−0.0140684	0.0202318	−0.695	0.48683
*ϕ*_4_	−0.0144893	0.0203167	−0.713	0.47574
*ϕ*_5_	−0.0104156	0.0202757	−0.514	0.60746
*ϕ*_6_	−0.0186630	0.0203238	−0.918	0.35847

**Table 3:** Mean (SD) of estimated ${\hat{\beta }}_{1}$s derived from frequentist GAMs.

*df*	$\sigma =0.5\hat{\sigma }$	$\sigma =0.6\hat{\sigma }$	$\sigma =0.7\hat{\sigma }$	$\sigma =0.8\hat{\sigma }$	$\sigma =0.9\hat{\sigma }$	$\sigma =\hat{\sigma }$
1	0.0095 (0.0018)	0.0095 (0.0022)	0.0094 (0.0026)	0.0094 (0.0029)	0.0094 (0.0033)	0.0094 (0.0037)
2	0.0091 (0.0018)	0.0091 (0.0022)	0.0091 (0.0025)	0.0091 (0.0029)	0.0091 (0.0033)	0.0090 (0.0037)
3	0.0068 (0.0019)	0.0068 (0.0022)	0.0068 (0.0026)	0.0067 (0.0030)	0.0067 (0.0034)	0.0067 (0.0038)
4	0.0056 (0.0019)	0.0056 (0.0023)	0.0056 (0.0026)	0.0056 (0.0030)	0.0056 (0.0034)	0.0055 (0.0038)
5	0.0064 (0.0019)	0.0064 (0.0022)	0.0064 (0.0026)	0.0064 (0.0030)	0.0064 (0.0034)	0.0064 (0.0038)
6	0.0065 (0.0019)	0.0065 (0.0023)	0.0065 (0.0027)	0.0065 (0.0031)	0.0065 (0.0034)	0.0064 (0.0038)
7	0.0059 (0.0019)	0.0059 (0.0023)	0.0058 (0.0027)	0.0058 (0.0031)	0.0058 (0.0035)	0.0058 (0.0039)
8	0.0051 (0.0020)	0.0051 (0.0023)	0.0050 (0.0027)	0.0050 (0.0032)	0.0050 (0.0035)	0.0050 (0.0040)
9	0.0049 (0.0020)	0.0049 (0.0024)	0.0049 (0.0028)	0.0049 (0.0032)	0.0049 (0.0036)	0.0048 (0.0040)
10	0.0049 (0.0020)	0.0049 (0.0024)	0.0048 (0.0028)	0.0048 (0.0032)	0.0048 (0.0036)	0.0048 (0.0040)
11	0.0049 (0.0020)	0.0048 (0.0024)	0.0048 (0.0028)	0.0048 (0.0032)	0.0048 (0.0036)	0.0048 (0.0040)
12	0.0048 (0.0020)	0.0048 (0.0024)	0.0048 (0.0028)	0.0048 (0.0032)	0.0047 (0.0036)	0.0047 (0.0041)
13	0.0048 (0.0020)	0.0048 (0.0024)	0.0048 (0.0028)	0.0048 (0.0032)	0.0047 (0.0036)	0.0047 (0.0040)
14	0.0049 (0.0020)	0.0048 (0.0024)	0.0048 (0.0028)	0.0048 (0.0032)	0.0048 (0.0036)	0.0047 (0.0041)
15	0.0049 (0.0020)	0.0049 (0.0024)	0.0048 (0.0028)	0.0048 (0.0032)	0.0048 (0.0036)	0.0048 (0.0041)
16	0.0049 (0.0020)	0.0049 (0.0024)	0.0048 (0.0028)	0.0048 (0.0032)	0.0048 (0.0036)	0.0048 (0.0041)
17	0.0049 (0.0020)	0.0049 (0.0024)	0.0048 (0.0028)	0.0048 (0.0032)	0.0048 (0.0036)	0.0048 (0.0041)
18	0.0049 (0.0020)	0.0048 (0.0024)	0.0048 (0.0028)	0.0048 (0.0033)	0.0048 (0.0037)	0.0047 (0.0041)
19	0.0049 (0.0020)	0.0048 (0.0024)	0.0048 (0.0028)	0.0048 (0.0033)	0.0048 (0.0037)	0.0047 (0.0041)
20	0.0049 (0.0020)	0.0048 (0.0025)	0.0048 (0.0029)	0.0048 (0.0033)	0.0048 (0.0037)	0.0047 (0.0042)

SD, standard deviation; GAM, generalized additive model.

**Table 4:** Mean (SD) of estimated ${\hat{\beta }}_{1}$s derived from Bayesian GAMs.

*df*	$\sigma =0.5\hat{\sigma }$	$\sigma =0.6\hat{\sigma }$	$\sigma =0.7\hat{\sigma }$	$\sigma =0.8\hat{\sigma }$	$\sigma =0.9\hat{\sigma }$	$\sigma =\hat{\sigma }$
1	0.0091 (0.0019)	0.0090 (0.0023)	0.0090 (0.0027)	0.0089 (0.0031)	0.0088 (0.0035)	0.0087 (0.0039)
2	0.0093 (0.0019)	0.0094 (0.0022)	0.0094 (0.0026)	0.0094 (0.0030)	0.0095 (0.0034)	0.0095 (0.0038)
3	0.0069 (0.0016)	0.0069 (0.0020)	0.0069 (0.0023)	0.0070 (0.0027)	0.0070 (0.0031)	0.0070 (0.0034)
4	0.0059 (0.0019)	0.0059 (0.0023)	0.0060 (0.0026)	0.0060 (0.0030)	0.0060 (0.0034)	0.0061 (0.0038)
5	0.0062 (0.0018)	0.0062 (0.0021)	0.0062 (0.0025)	0.0061 (0.0028)	0.0061 (0.0032)	0.0060 (0.0036)
6	0.0066 (0.0019)	0.0067 (0.0023)	0.0067 (0.0027)	0.0067 (0.0031)	0.0068 (0.0035)	0.0068 (0.0039)
7	0.0062 (0.0017)	0.0062 (0.0021)	0.0063 (0.0024)	0.0064 (0.0028)	0.0064 (0.0032)	0.0065 (0.0036)
8	0.0053 (0.0018)	0.0054 (0.0021)	0.0054 (0.0025)	0.0055 (0.0029)	0.0056 (0.0032)	0.0056 (0.0036)
9	0.0054 (0.0020)	0.0055 (0.0024)	0.0056 (0.0028)	0.0057 (0.0031)	0.0058 (0.0035)	0.0058 (0.0039)
10	0.0049 (0.0019)	0.0049 (0.0023)	0.0049 (0.0028)	0.0050 (0.0032)	0.0050 (0.0036)	0.0050 (0.0040)
11	0.0046 (0.0018)	0.0045 (0.0022)	0.0045 (0.0026)	0.0044 (0.0029)	0.0043 (0.0033)	0.0043 (0.0037)
12	0.0049 (0.0018)	0.0049 (0.0022)	0.0049 (0.0026)	0.0049 (0.0030)	0.0049 (0.0034)	0.0049 (0.0038)
13	0.0051 (0.0018)	0.0052 (0.0022)	0.0052 (0.0025)	0.0053 (0.0029)	0.0053 (0.0032)	0.0054 (0.0036)
14	0.0047 (0.0017)	0.0047 (0.0021)	0.0047 (0.0024)	0.0047 (0.0028)	0.0046 (0.0032)	0.0046 (0.0036)
15	0.0050 (0.0019)	0.0050 (0.0023)	0.0050 (0.0027)	0.0051 (0.0031)	0.0051 (0.0035)	0.0051 (0.0039)
16	0.0047 (0.0021)	0.0047 (0.0025)	0.0046 (0.0030)	0.0046 (0.0034)	0.0045 (0.0039)	0.0045 (0.0044)
17	0.0048 (0.0020)	0.0048 (0.0025)	0.0048 (0.0029)	0.0048 (0.0033)	0.0048 (0.0037)	0.0047 (0.0042)
18	0.0048 (0.0017)	0.0048 (0.0020)	0.0048 (0.0024)	0.0048 (0.0027)	0.0047 (0.0031)	0.0048 (0.0034)
19	0.0052 (0.0023)	0.0052 (0.0028)	0.0053 (0.0032)	0.0054 (0.0037)	0.0054 (0.0042)	0.0055 (0.0047)
20	0.0049 (0.0018)	0.0049 (0.0021)	0.0049 (0.0025)	0.0049 (0.0028)	0.0049 (0.0032)	0.0049 (0.0036)

SD, standard deviation; GAM, generalized additive model.

**Table 5:** Mean (SD) of estimated ${\hat{\beta }}_{1}$s derived from Bayesian GAMs with different priors.

µ(*β*_1_)	$\mathrm{Var}(\beta_{1})=0.5\beta_{1}$	$\mathrm{Var}(\beta_{1})=0.6\beta_{1}$	$\mathrm{Var}(\beta_{1})=0.7\beta_{1}$	$\mathrm{Var}(\beta_{1})=0.8\beta_{1}$	$\mathrm{Var}(\beta_{1})=0.9\beta_{1}$	$\mathrm{Var}(\beta_{1})=\beta_{1}$
0.001	0.0048 (0.0018)	0.0048 (0.0018)	0.0048 (0.0018)	0.0048 (0.0018)	0.0048 (0.0018)	0.0048 (0.0018)
0.002	0.0046 (0.0020)	0.0046 (0.0020)	0.0046 (0.0020)	0.0046 (0.0020)	0.0046 (0.0020)	0.0046 (0.0020)
0.003	0.0052 (0.0018)	0.0052 (0.0018)	0.0052 (0.0018)	0.0052 (0.0018)	0.0052 (0.0018)	0.0052 (0.0018)
0.004	0.0045 (0.0018)	0.0045 (0.0018)	0.0045 (0.0018)	0.0045 (0.0018)	0.0045 (0.0018)	0.0045 (0.0018)
0.005	0.0049 (0.0020)	0.0050 (0.0020)	0.0050 (0.0020)	0.0049 (0.0020)	0.0049 (0.0020)	0.0049 (0.0020)
0.006	0.0048 (0.0018)	0.0048 (0.0018)	0.0048 (0.0018)	0.0048 (0.0018)	0.0048 (0.0018)	0.0048 (0.0018)
0.007	0.0047 (0.0018)	0.0047 (0.0018)	0.0047 (0.0018)	0.0047 (0.0018)	0.0047 (0.0018)	0.0047 (0.0018)
0.008	0.0048 (0.0018)	0.0048 (0.0018)	0.0048 (0.0018)	0.0048 (0.0018)	0.0048 (0.0018)	0.0048 (0.0018)
0.009	0.0048 (0.0020)	0.0048 (0.0020)	0.0048 (0.0020)	0.0048 (0.0020)	0.0048 (0.0020)	0.0048 (0.0020)
0.010	0.0046 (0.0020)	0.0046 (0.0020)	0.0046 (0.0020)	0.0046 (0.0020)	0.0046 (0.0020)	0.0046 (0.0020)
0.011	0.0047 (0.0018)	0.0047 (0.0018)	0.0047 (0.0018)	0.0047 (0.0018)	0.0047 (0.0018)	0.0047 (0.0018)
0.012	0.0051 (0.0017)	0.0051 (0.0017)	0.0051 (0.0017)	0.0051 (0.0017)	0.0051 (0.0017)	0.0051 (0.0017)
0.013	0.0046 (0.0016)	0.0046 (0.0016)	0.0046 (0.0016)	0.0046 (0.0016)	0.0046 (0.0016)	0.0046 (0.0016)
0.014	0.0050 (0.0019)	0.0050 (0.0019)	0.0050 (0.0019)	0.0050 (0.0019)	0.0050 (0.0019)	0.0050 (0.0019)
0.015	0.0050 (0.0020)	0.0050 (0.0020)	0.0050 (0.0020)	0.0050 (0.0020)	0.0050 (0.0020)	0.0050 (0.0020)
0.016	0.0046 (0.0018)	0.0046 (0.0018)	0.0046 (0.0018)	0.0046 (0.0018)	0.0046 (0.0018)	0.0046 (0.0018)
0.017	0.0049 (0.0020)	0.0049 (0.0020)	0.0049 (0.0020)	0.0049 (0.0020)	0.0049 (0.0020)	0.0049 (0.0020)
0.018	0.0050 (0.0017)	0.0050 (0.0017)	0.0050 (0.0017)	0.0050 (0.0017)	0.0050 (0.0017)	0.0050 (0.0017)
0.019	0.0049 (0.0016)	0.0049 (0.0016)	0.0049 (0.0016)	0.0049 (0.0016)	0.0049 (0.0016)	0.0049 (0.0016)
0.020	0.0051 (0.0018)	0.0051 (0.0018)	0.0051 (0.0018)	0.0051 (0.0018)	0.0051 (0.0018)	0.0051 (0.0018)

SD, standard deviation; GAM, generalized additive model.

**Table 6:** Mean (SD) of estimated ${\hat{\beta }}_{1}$s derived from Bayesian GAMs of different true *β*_1_s.

True *β*_1_	$\mathrm{Var}(\beta_{1})=0.5\beta_{1}$	$\mathrm{Var}(\beta_{1})=0.6\beta_{1}$	$\mathrm{Var}(\beta_{1})=0.7\beta_{1}$	$\mathrm{Var}(\beta_{1})=0.8\beta_{1}$	$\mathrm{Var}(\beta_{1})=0.9\beta_{1}$	$\mathrm{Var}(\beta_{1})=\beta_{1}$
0.001	0.0008 (0.0018)	0.0008 (0.0018)	0.0008 (0.0018)	0.0008 (0.0018)	0.0008 (0.0018)	0.0008 (0.0018)
0.002	0.0016 (0.0020)	0.0016 (0.0020)	0.0016 (0.0020)	0.0016 (0.0020)	0.0016 (0.0020)	0.0016 (0.0020)
0.003	0.0032 (0.0018)	0.0032 (0.0018)	0.0032 (0.0018)	0.0032 (0.0018)	0.0032 (0.0018)	0.0032 (0.0018)
0.004	0.0035 (0.0018)	0.0035 (0.0018)	0.0035 (0.0018)	0.0035 (0.0018)	0.0035 (0.0018)	0.0035 (0.0018)
0.005	0.0049 (0.0020)	0.0050 (0.0020)	0.0050 (0.0020)	0.0049 (0.0020)	0.0049 (0.0020)	0.0049 (0.0020)
0.006	0.0058 (0.0018)	0.0058 (0.0018)	0.0058 (0.0018)	0.0058 (0.0018)	0.0058 (0.0018)	0.0058 (0.0018)
0.007	0.0067 (0.0018)	0.0067 (0.0018)	0.0067 (0.0018)	0.0067 (0.0018)	0.0067 (0.0018)	0.0067 (0.0018)
0.008	0.0078 (0.0018)	0.0078 (0.0018)	0.0078 (0.0018)	0.0078 (0.0018)	0.0078 (0.0018)	0.0078 (0.0018)
0.009	0.0088 (0.0020)	0.0088 (0.0020)	0.0088 (0.0020)	0.0088 (0.0020)	0.0088 (0.0020)	0.0088 (0.0020)
0.010	0.0096 (0.0020)	0.0096 (0.0020)	0.0096 (0.0020)	0.0096 (0.0020)	0.0096 (0.0020)	0.0096 (0.0020)
0.011	0.0107 (0.0018)	0.0107 (0.0018)	0.0107 (0.0018)	0.0107 (0.0018)	0.0107 (0.0018)	0.0107 (0.0018)
0.012	0.0121 (0.0017)	0.0121 (0.0017)	0.0121 (0.0017)	0.0121 (0.0017)	0.0121 (0.0017)	0.0121 (0.0017)
0.013	0.0125 (0.0016)	0.0125 (0.0016)	0.0126 (0.0016)	0.0126 (0.0016)	0.0126 (0.0016)	0.0126 (0.0016)
0.014	0.0140 (0.0019)	0.0140 (0.0019)	0.0140 (0.0019)	0.0140 (0.0019)	0.0140 (0.0019)	0.0140 (0.0019)
0.015	0.0150 (0.0020)	0.0150 (0.0020)	0.0150 (0.0020)	0.0150 (0.0020)	0.0150 (0.0020)	0.0150 (0.0020)
0.016	0.0156 (0.0019)	0.0156 (0.0019)	0.0156 (0.0019)	0.0156 (0.0019)	0.0156 (0.0019)	0.0156 (0.0019)
0.017	0.0170 (0.0020)	0.0170 (0.0020)	0.0170 (0.0020)	0.0170 (0.0020)	0.0170 (0.0020)	0.0170 (0.0020)
0.018	0.0180 (0.0017)	0.0180 (0.0017)	0.0180 (0.0017)	0.0180 (0.0017)	0.0180 (0.0017)	0.0180 (0.0017)
0.019	0.0189 (0.0017)	0.0189 (0.0017)	0.0189 (0.0017)	0.0189 (0.0017)	0.0189 (0.0017)	0.0190 (0.0017)
0.020	0.0201 (0.0019)	0.0201 (0.0019)	0.0202 (0.0019)	0.0202 (0.0019)	0.0202 (0.0019)	0.0202 (0.0019)

SD, standard deviation; GAM, generalized additive model.

## Data Availability

Data sharing is not applicable to this article as datasets were simulated during the current study. All the parameters for generating the simulated datasets were provided in Table 1 and Table 2.

## Abbreviations

AQM	Air quality monitoring
CDR	Causes of Death Registry
CrI	Credible interval
*df*	Degrees of freedom
DOW	Day of week
GAM	Generalized additive model
GLM	Generalized linear model
ICD-10	International Classification of Diseases Codes, version 10
IQR	Interquartile range
LOESS	locally weighted smoothers
MCMC	Markov Chain Monte Carlo
PM	Particulate matter
PM_2.5_	Fine particles with a diameter of 2.5 μm or less
SCDC	Shanghai Municipal Center for Disease Control and Prevention
SD	Standard deviation
SE	Standard error
SWT	Synoptic weather types
VCM	Varying coefficient model
